# Traffic medicine–related research: a scientometric analysis

**DOI:** 10.1186/1471-2458-13-541

**Published:** 2013-06-05

**Authors:** Beatrix Groneberg-Kloft, Doris Klingelhoefer, Simona E Zitnik, Cristian Scutaru

**Affiliations:** 1 Otto-Heubner-Center, Charité-Universitätsmedizin Berlin, Free University Berlin and Humboldt-University, Berlin, Germany; 2 Institute of Occupational Medicine, Social Medicine and Environmental Medicine, Goethe-University, Frankfurt am Main, Germany; 3 Institute of Occupational Medicine, Charité-Universitätsmedizin Berlin, Free University Berlin and Humboldt-University, Berlin, Germany

## Abstract

**Objective:**

Traffic crashes and related injuries are important causes of morbidity and mortality and impose insofar an important burden on public health. However, research in this area is often under-funded. The aim of this study was to analyse quantity, evolution and geographic distribution of traffic medicine-related research. This multi-sectorial field covers both transport and health care sectors.

**Design:**

A scientometric approach in combination with visualizing density equalizing mapping was used to analyse published data related to the field of traffic medicine between 1900 and 2008 within the “Web of Science” (WoS) database.

**Results:**

In total, 5,193 traffic medicine-associated items were produced between 1900 and 2008. The United States was found to have the highest research activity with a production of n = 2,330 published items, followed by Germany (n = 298) and Canada (n = 219). Cooperation analyses resulted in a peak of published multilateral cooperations in the year of 2003. The country with the highest multilateral activity was the USA. The average number of cited references per publication varied heavily over the last 20 years with a maximum of 27.67 in 1995 and a minimum of 15.08 in 1998. Also, a further in-depth analysis was performed with a focus solely on public health aspects which revealed similar trends.

**Conclusions:**

Summarizing the present data it can be stated traffic medicine-related research productivity grows annually. Also, an active networking between countries is present. The data of the present study may be used by scientific organisations in order to gain detailed information about research activities in this field which is extremely important for public health.

## Introduction

Traffic crashes and related injuries belong to the most important anthropogenic causes of morbidity and mortality in all parts of the world [1-3]. They are therefore a major burden of disease that affects issues of public health. A greater proportion of the burden of injury and disability is found in developing countries, due to the improved safety in industrialized countries [4]. In detail, road crashes, with 50 million people being injured, account for approximately 1.2 million deaths each year. Road traffic injuries have been reported to be the second leading cause of death among young people in developing countries aged 15 – 29 years and among children aged 5 – 14 years [5,6]. It can therefore be assumed that all means to increase traffic safety may help to reduce the global burden of disease. Studies analysing the potential effect of an absence of concerted efforts at prevention have concluded that there might be a dramatic increase in mortality and morbidity. It was calculated that road traffic deaths would increase from the 10th position in the list of the top 24 causes of death in 2002 to position eight by 2030 if concerted efforts at the prevention of road crashes are not implemented [7].

The last 50 years can be characterized by an exponential increase in medical, social and technical research in general [8,9]. To deal with the large amount of data, quantitative and qualitative assessment tools for scientific output have become increasingly important. They now play a key role in the allocation of funding and prioritisation of resources as being demonstrated by the Research Assessment Exercise in the UK [10].

Despite its afore mentioned disease burden caused by traffic crashes, relatively little effort has previously been made to understand the trends emanating from traffic medicine-associated literature on a bibliographical basis. One reason is probably the multi-sectorial nature of this area which covers both transport and health care sectors. While there has been some concentration on the bibliometrics of occupational medicine generally [11-16], only little is known about specific scientific output related to traffic. Thus, the project “New Quality and Quantity Indices in Science” (NewQIS) [17,18] decided to perform a detailed analysis of research yield in traffic medicine from 1900 to 2008. We applied large-scale data analysis, bibliometric indicators of production and quality, and density-equalizing calculations. These calculations are based on a algorithm that was published by Gastner and Newman [19]. The mappings are used to illustrate international correlations by resizing single countries in proportion to a defined variable such as article number or citation number.

## Methods

### Data source

The present study was integrated into the NewQIS project. In this project, bibliometric tools are combined to visualizing techniques [17,18]. All analyzed data were retrieved from Thomson Scientifics online database “Web of Science” (WoS). The PubMed database lacks citation information of the publications. This is why the entire analysis was conducted by the means of the WoS database.

### Time span

The time frame was limited to the period between 1900 and 2008. For this purpose, the “Change Limits and Settings” function was adjusted initially and the query was made under this presetting. The years 2009–2012 were excluded in order to focus the study exclusively on completed years.

### Data categorization

Data categorization was performed as previously described [20]. In brief, the WoS database provided several tools to analyze entries according to specified parameters. The data set was analyzed by means of publication country, publication year, publishing author, publishing journals and published document type. Multiple distributions led to higher publication numbers when adding up results after analysis; for example, when a super regional publication is distributed to several countries. A common data processing program was used to display the results in tables, charts and diagrams.

### Search strategies

Two search strategies were employed: For the general query (termed “general search”), the terms "traffic* securit*", "car crash*", "car accident*", "transport* accident*", "transport* crash*", "traffic* safet*", "car safet*", "vehicle crash*", "vehicle safet*" were connected with the Boolean operator “OR” and entered in the search field “Topic”. The asterisk stands as a placeholder for different word variations. In a second query that specifically addressed the area of public health and traffic, the terms “*public health*” AND “*transport*” OR “*public health*” and “*traffic*” were used. This search is termed “public health search”. This search was performed until the year 2011.

### Primary data

For a first insight, the total number of published items was recorded together with the type of publication (original article, meeting abstract, review etc.), assigned subject areas, and publishing journals.

### Citations per year

We assessed the total number of citations of all identified published items. Since they were all recorded until the date of analysis, an average citation per item was computed for those years with at least 30 published items.

### Analysis of origin / language

The information about the address of the authors was analyzed in order to determine the country of origin of the articles. For countries such as the former USSR which split up, the current location of the city (country) was taken into account when determining the origin of the publication.

### Citation characteristics - average citation rate (countries)

For all countries with at least 30 published items the average citation rate was calculated. For a better understanding of the country-specific citation characteristics, a modified H-Index for countries was used. In this respect, the theory of the H-Index (Hirsch-Index) was extrapolated on all articles originating from a given country. Hence, the definition of this country-specific H-Index is: A country has index “h” if “h” of its “N_p_” papers have at least “h” citations each, and the other (“N_p_ - h”) papers have no more than “h” citations each.

### Publication type

All publications were analysed under following aspects:

• Number of published items

• Total number of citations

• Average number of citations

Assuming that the page format (i.e. the number of characters per page) of the journals is rather identical, the length of the publications was computed giving the start and end page. This procedure was performed for years in which 30 published items with page information was present.

### Citation quantities

Identified publications were also analyzed by means of citation. Therefore, the feature “Citation Report” was used to calculate the citation rate of both authors and citations per year of citation. The complete “Citation Report” was downloaded and by special VBA Software further analyzed and than edited with “Microsoft Excel”. Afterwards, the 10 most productive authors were analysed for citations. The average citation rate was defined as the quotient of the total citation number divided by the publications listed for the author in question.

### Density equalizing mapping

This study used density equalizing mapping in order to illustrate different parameters. The principles of the underlying calculations were reported by Gastner and Newman [19] and integrated into the NewQIS platform projects [21]. Software using the method of density equalizing mapping was employed to determine international correlations. This method resized countries proportionally according to a predefined variable. The territory with the highest number of publications was depicted largest on the associated map.

## Results

### Number of published items

**Figure 1:**
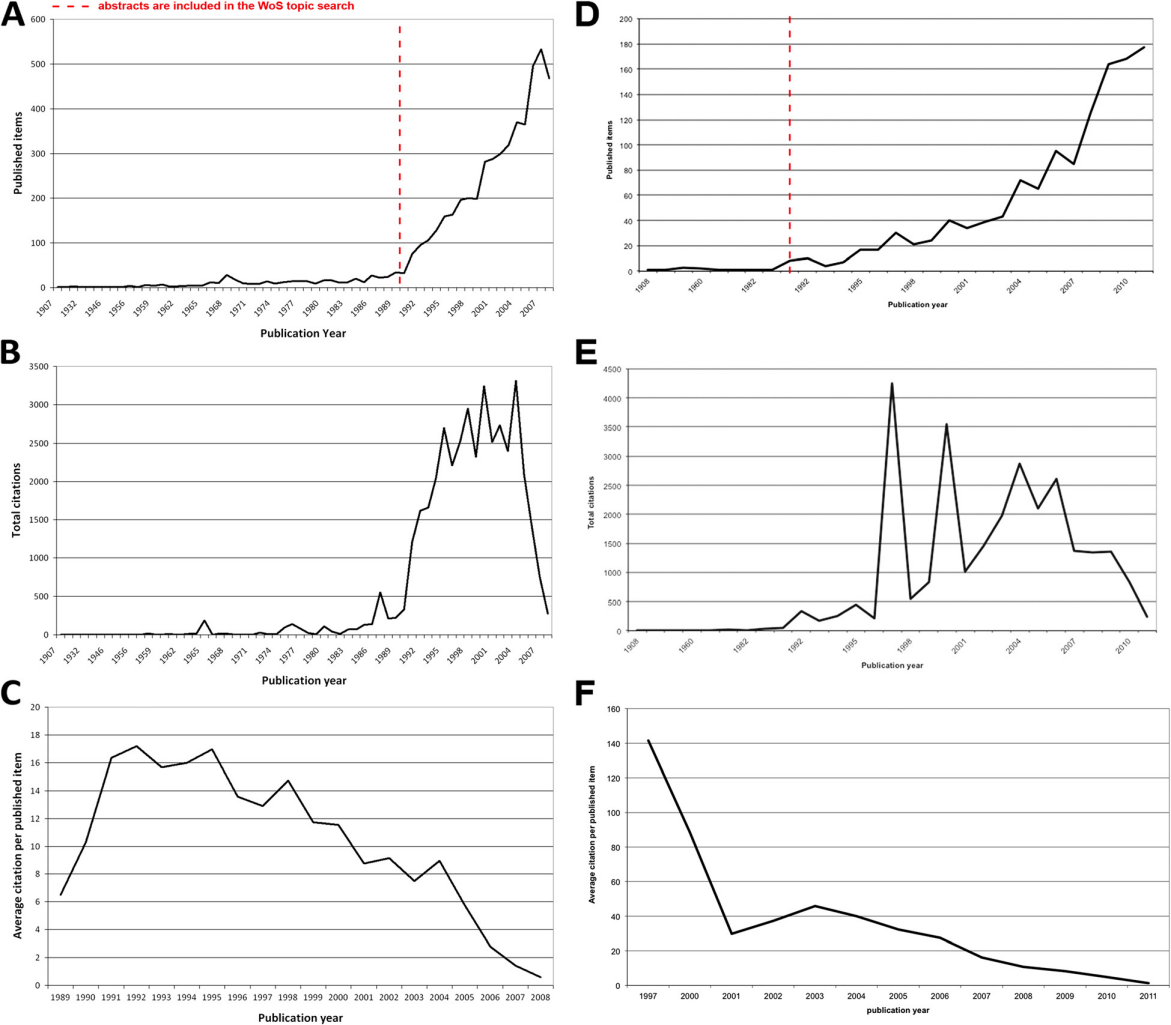
**General parameters A** General search: Evolution of the number of published items over the time period **B** General search: Evolution of the total number of citations for the time period **C** General search: Evolution of the average citation per item **D** Public health search: Evolution of the number of published items over the time period **E** Public health search: Evolution of the total number of citations for the time period **F** Public health search: Evolution of the average citation per item.

The general search query achieved 5,193 results. The oldest work was published 1907. The highest number of publications was published in 2007 (533), followed by 2006 (495) and 2008 (467) (Figure 1A). Starting with 1990 we found a dramatic increase in the number of published items. In the public health search that narrowly focused on studies with a direct link to public health and transport/traffic medicine due to the use of the search terms “*public health*” AND “*transport*” OR “*public health*” and “*traffic*”, a total of 1257 publications were found until 2011 with a peak of 177 published items in this year (Figure 1D).

### Citation information

Looking at the evolution of citations in the general search, we found a similar evolution, with a very strong increase after 1990, however after 2004 the number of total citations dropped dramatically. The reason for this is that the articles were “too new” and there was no time for citing them so far. Publications published 2004 reached the highest number of citations (3314), followed by 2000 (3241) and 1998 (2949) (Figure 1B). Analyzing the average citation per item for years in which at least 30 publications were issued, we found a descending trend starting with 1992 which holds the highest value of 17.19 citations per item (Figure 1C). In the public health search, citation peaks were found in 1997, 2000 and 2004 (Figure 1F).

### Evolution of the average number of authors per published item

**Figure 2:**
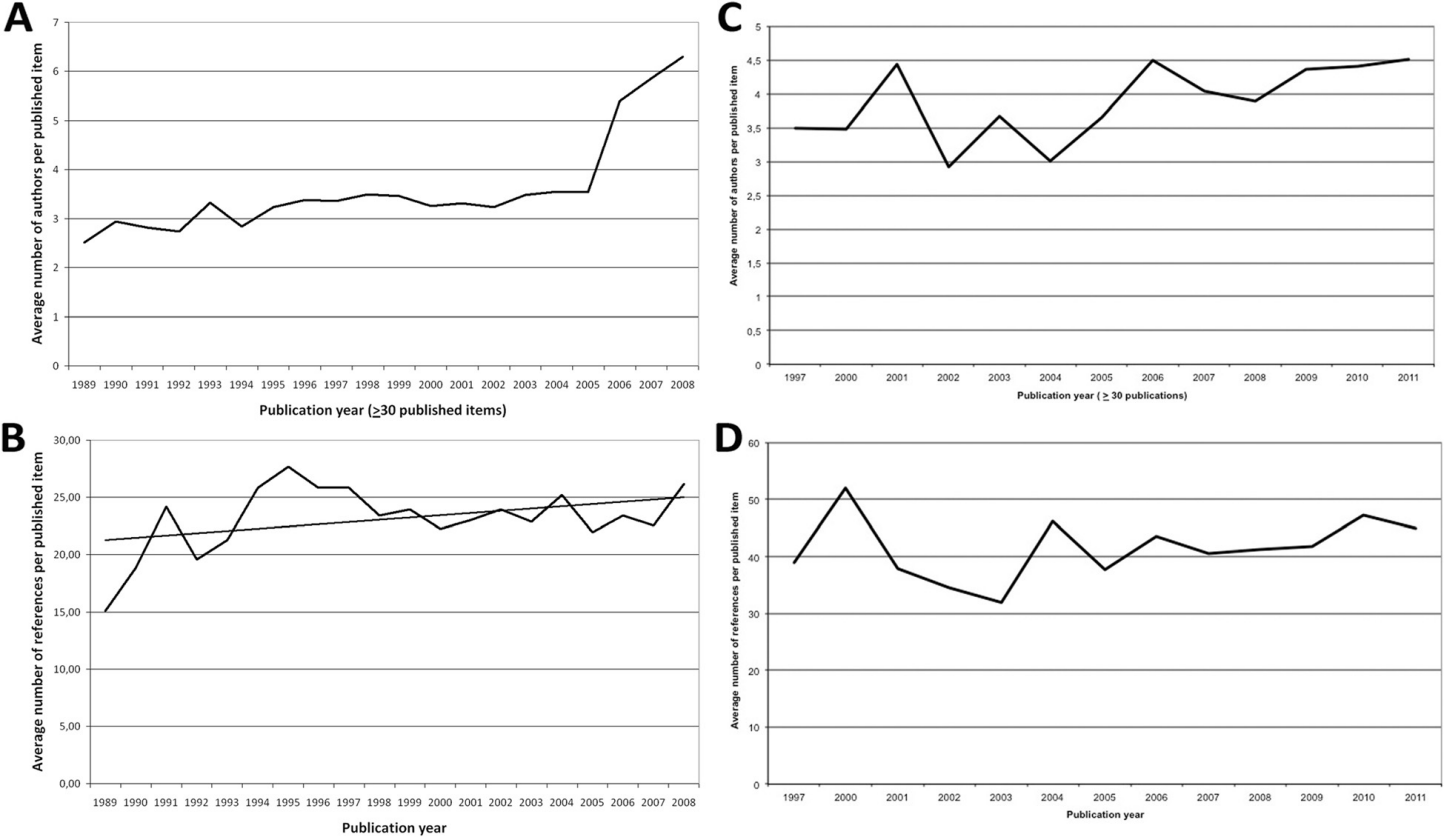
**Evolutionary aspects A** General search: Evolution of the average number of authors per published item **B** General search: Evolution of the average number of cited references **C** Public health search: Evolution of the average number of authors per published item **D** Public health search: Evolution of the average number of cited references.

The average number of authors per published item was computed for years in which at least 30 published items appeared. For the general search, the highest value was calculated for 2008 (6.29) followed by 2007 (5.85) and 2006 (5.40). For the other years this value was relatively constant varying between 2.52 and 3.55 (Figure 2A). For the public health search, the average number of authors varied between 3 and 4.5 Figure 2C).

### Evolution of the cited references

The average number of cited references per article varied in the general search over the last 20 years between a maximum of 27 in 1995 and a minimum of 15 in 1998 with a slight increasing tendency (Figure 2B). In the public health search, the average number varied between 31 and 52 (Figure 2D).

### Language related analysis

In the general search 95.22% of all analyzed publications were written in English. The second language by number of publications was German with only 2.87% followed by French (0.83%), Spanish (0.80%) and other (0.67%). All other languages with less than 10 publications were Russian (7), Portuguese (6), Japanese and Norwegian (each 4), Turkish (3), Chinese (2), and Croatian, Czech, Dutch, Italian, Lithuanian, Polish, Romanian, Slovenian and Swedish each with 1 publication. Most of the analyzed publications in the general search were original articles (60.37%) followed by proceedings papers (27.40%), editorial material (2.95%), reviews (2.25%), letters (1.77%), book reviews (1.12%), notes (0.94%), news items (0.35%) and reprints (0.21%).

### Country-related analysis

#### Published items

**Figure 3:**
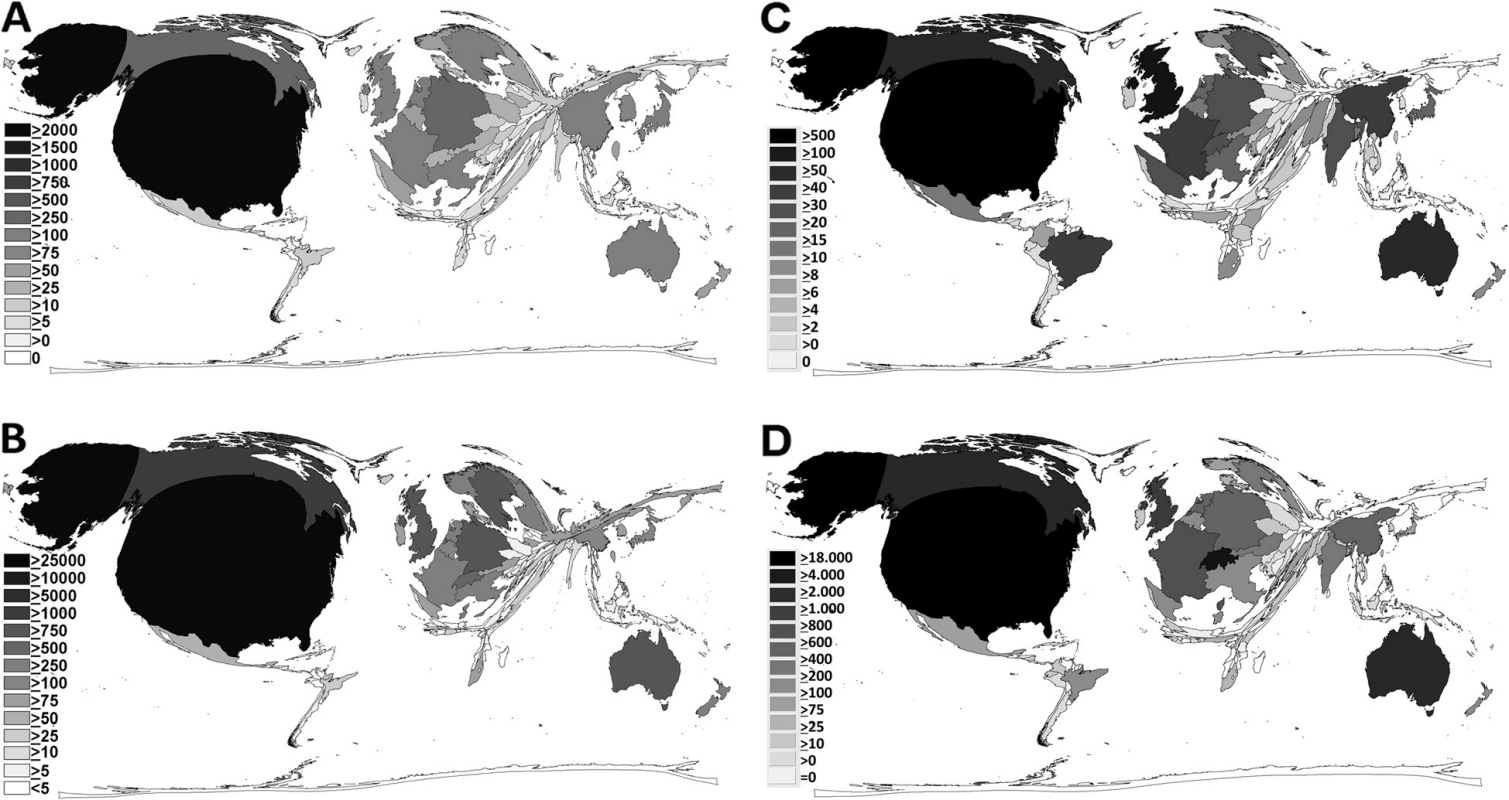
**Density equalizing mapping A** General search: Distribution of published items **B** General search: Distribution of total citations **C** Public health search: Distribution of published items **D** Public health search: Distribution of total citations.

In the general search a total of 87 different countries were identified as source for the 4,775 publications. 418 publications had no information concerning the origin of the authors. The USA was with a total of 2,330 published items the country with the highest output, followed by Germany (298), Canada (265), UK (219) and Australia (191). From the corresponding density equalizing cartogram it can be observed that North America, Europe, Australia and Eastern Asia concentrated the great majority of all published items, with very little contributions from Africa, Asia, and South America (Figure 3A). For the public health search, slight differences in the proportions were present, i.e. with Australia playing a more prominent role (Figure 3C).

#### Total citation analysis

The analysis of the total number of citations per country revealed a slightly different line-up, with the USA still unreachable with a total of 26,426 citations, followed by Canada (2,841), UK (2,165), Australia (1,886) and Sweden (1,231) (Figure 3B). When comparing the corresponding density equalizing cartograms, differences in the proportions could be shown between the general search (Figure 3B) and the public health search (Figure 3D); i.e. Australia and European countries play a more prominent role.

#### Average citation per item

**Figure 4:**
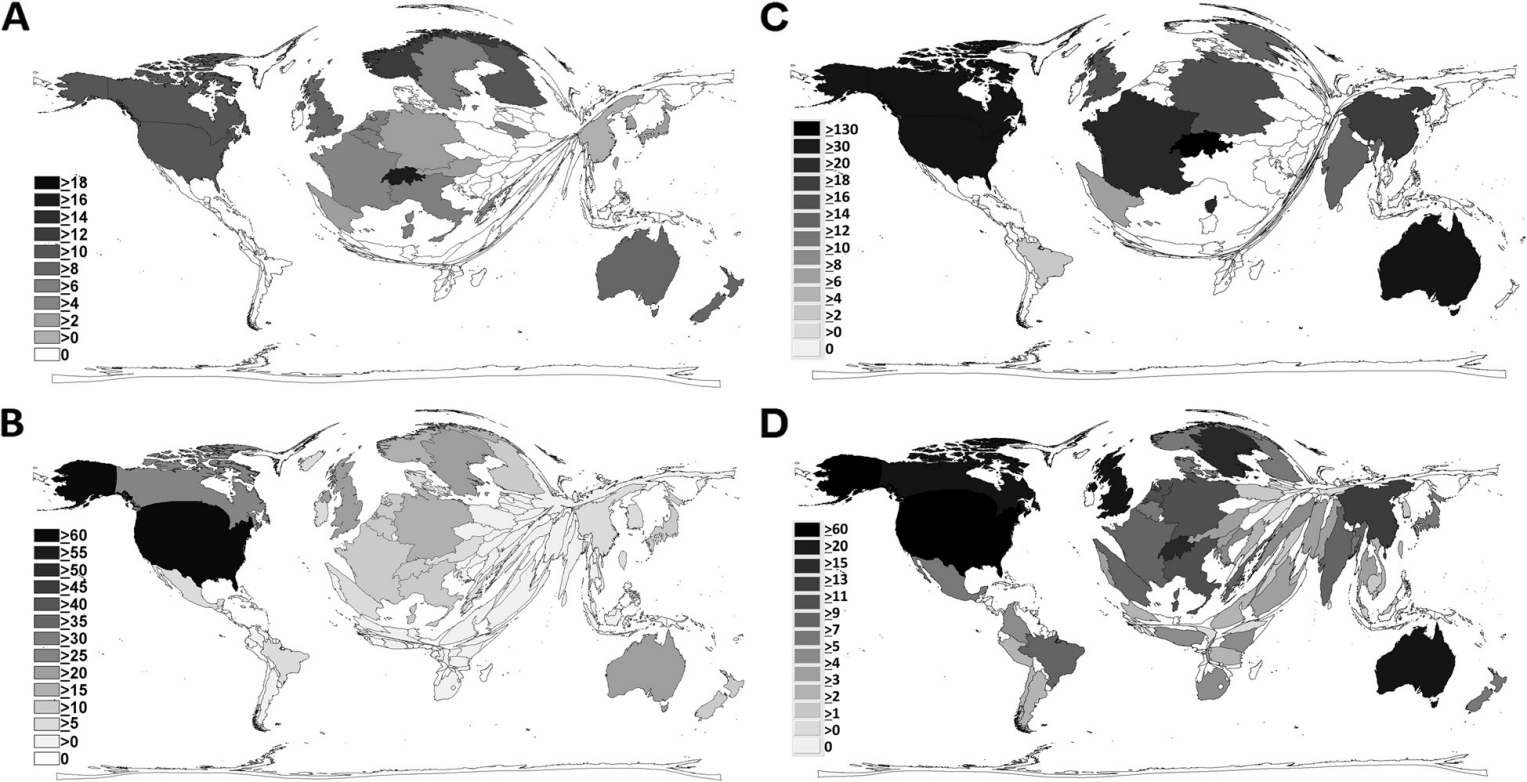
**Density equalizing mapping and advanced indices A** General search: Average citation per published item **B** General search: Modified H-Index of countries **C** Public health search: Average citation per published item **D** Public health search: Modified H-Index of countries.

Computing the average citation per item for countries with at least 30 published items, a completely different situation was present in the general search with Switzerland first with an average of 17.96 citations per item, followed by Norway (12.20), USA (11.34), Finland (11.11) and Canada (10.72) (Figure 4A). In the public health search becomes a slightly different picture apparent (Figure 4C).

#### Modified H-Index

The analysis of the modified H-Index for the countries resulted in the general search with the USA being the leading country with a value of 62, followed by Canada (29) (Figure 4B). In the public health search, again, other countries including European countries and Australia bridged the gap which can be visualized by the density equalizing mapping (Figure 4D).

#### International cooperation between countries

**Figure 5:**
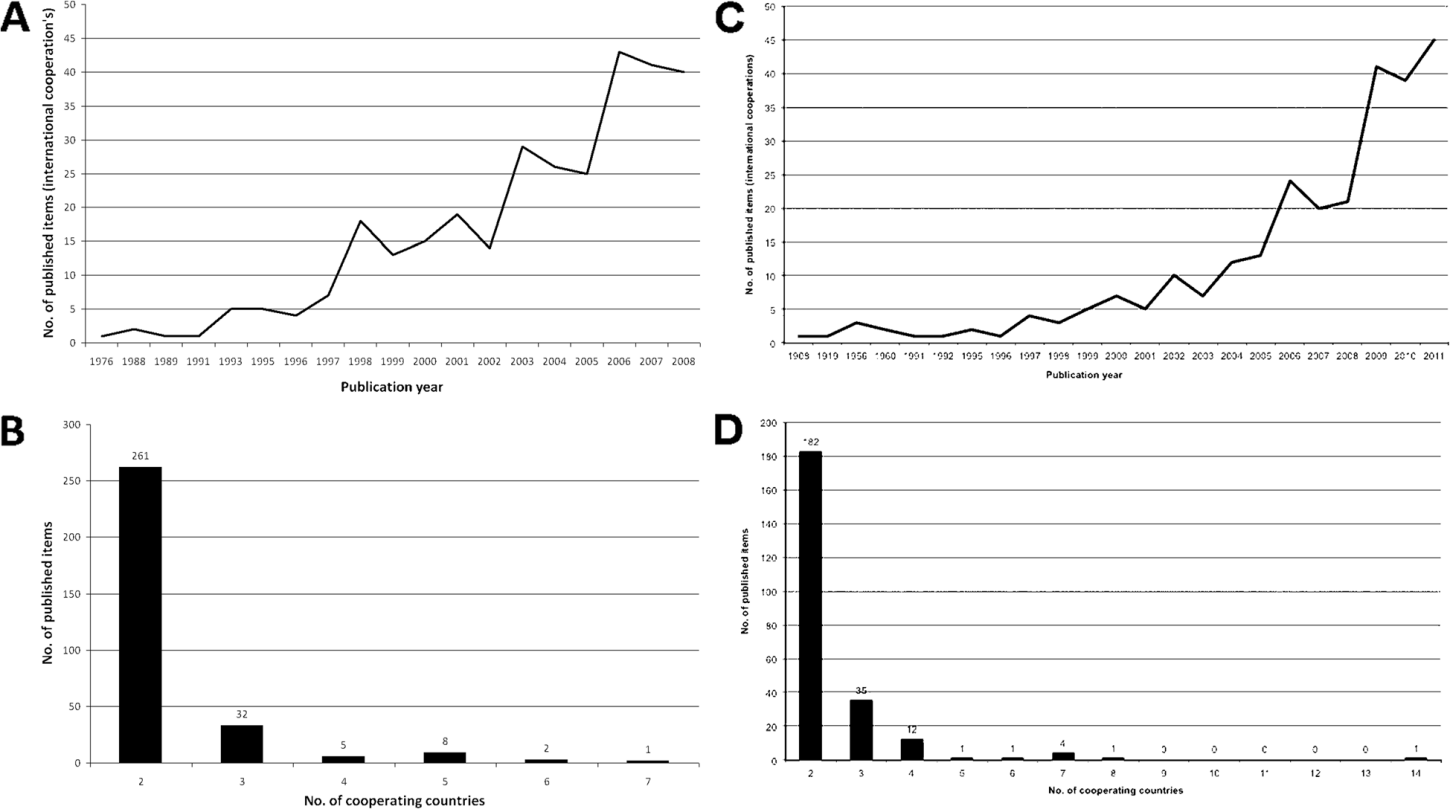
**Cooperations A** General search: Evolution of international cooperation **B** General search: Distribution of the cooperation articles by the number of cooperating Countries **C** Public health search: Evolution of international cooperation **D** Public health search: Distribution of the cooperation articles by the number of cooperating Countries.

Out of the 5,193 published items within the general search, 309 were the result of international cooperations. From the 87 countries identified in the general search, 69 are involved in international cooperative works. The first international cooperation article was written in 1976. Since then a steady increase in the number of teamwork was present. In 2003 the highest number (43) of international cooperation articles was found, followed by 2007 (41) and 2008 (40) (Figure 5A). In the narrowly focussed public health search, 268 items were published as international cooperation with an increasing tendency (Figure 5C). This is a three-fold higher percentage than in the general search (268/1257 = 21% vs. 309/5193 = 6%).

In the general search, the highest number of cooperation articles was a result of a bilateral liaisons (261 out of the 309), followed by team works between 3 countries (32), cooperations between 5 countries (8), cooperative works between 4 countries (5), cooperations between 6 countries (2) and finally 1 published item was the result of a collaboration between 7 different countries (Figure 5B). In the public health search, also the highest number of cooperations was due to a bilateral collaboration (182), followed by trilateral works (35) (Figure 5D).

**Figure 6:**
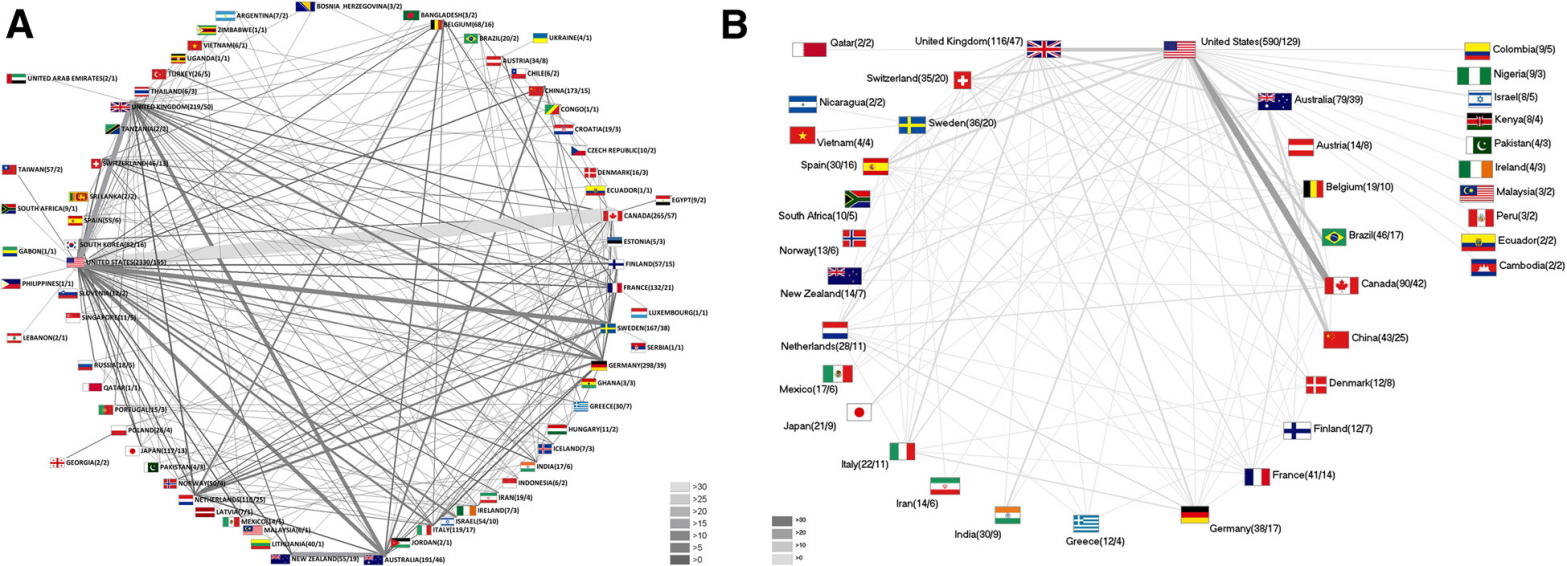
**International cooperation network A General search, B Public health search.** Greyscales and thickness of the bars code the amount of cooperation between two countries. The numbers behind the name of the country represent total number of articles, respectively total number of international cooperations.

The USA and Canada had the highest number of bilateral cooperation articles (44) in the general search, followed by cooperation between the USA and UK (17), Australia and New Zealand (16), USA and Sweden (15). Cooperations between the UK and Australia, the USA and Australia and the USA and South Korea were each 12 published items (Figure 6A). In the public health search, a slightly different chart distribution was present (Figure 6B).

### Institution analysis - cooperation between institutions

**Figure 7:**
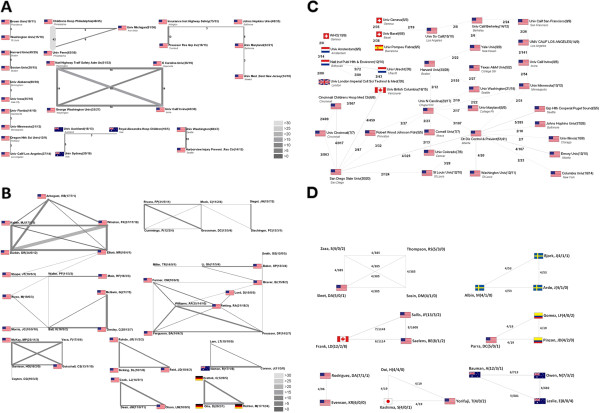
**Networking A General search: Cooperation network between institutions with at least 10 published items.** Threshold was set to 3 cooperating articles for improved readability **B** General search: Author cooperation network. Greyscale and thickness stand for amount of cooperation. The numbers behind the name are total number of published items / as first author / as senior author respectively. **C** Public health search: Cooperation network between institutions with at least 10 publi*s*hed items. Threshold was set to 2 cooperating articles for improved readability **D** Public health search: Author cooperation network. The numbers behind the name are total number of published items / as first author / as senior author respectively.

For all institutions with at least 10 published items a cooperation network chart analysis was computed. A total of 79 institutions out of the total of 3,965 identified reached this threshold in the general search. The Children’s Hospital Philadelphia and the University Penn from Philadelphia hold the highest number of cooperating articles (42). The second highest cooperation activity was present between the National Highway Traffic Safety Administration of US from Washington together with the University of California Irvine (17 published items). The only non-US cooperation which reached the threshold of 3 publications was formed by Australian institutions: University Sydney – University of Auckland (5), and University of Auckland – Royal Alexandra Hospital for Children (3) (Figure 7A). For the more narrowly focussed public health search, a different distribution was present with other institutions leading. However, the amount of cooperative works was relatively low, i.e. the highest amount of identified published cooperation articles was 5 (Figure 7C).

### Author analysis - cooperation network

Similarly to the Institution analysis, the cooperation network for all authors with at least 10 published items was computed. In the general search, the highest level of cooperation was reached by Durbin, DR and Winston, FK from the USA with 23 published items, followed by Richter, M and Otte, D from Germany with 17 publications and McGwin, G and Owsley, C from USA with 15 published items (Figure 7B). In the public health search, the highest number of identified cooperations was present between Sallis, JF and Salens, BE (8), and Sallis, JF and Frank, LD (7) (Figure 7D). It was not possible for some authors to retrieve the country affiliation because this information was not available in the WoS database.

### Subject area analysis - published items

**Figure 8:**
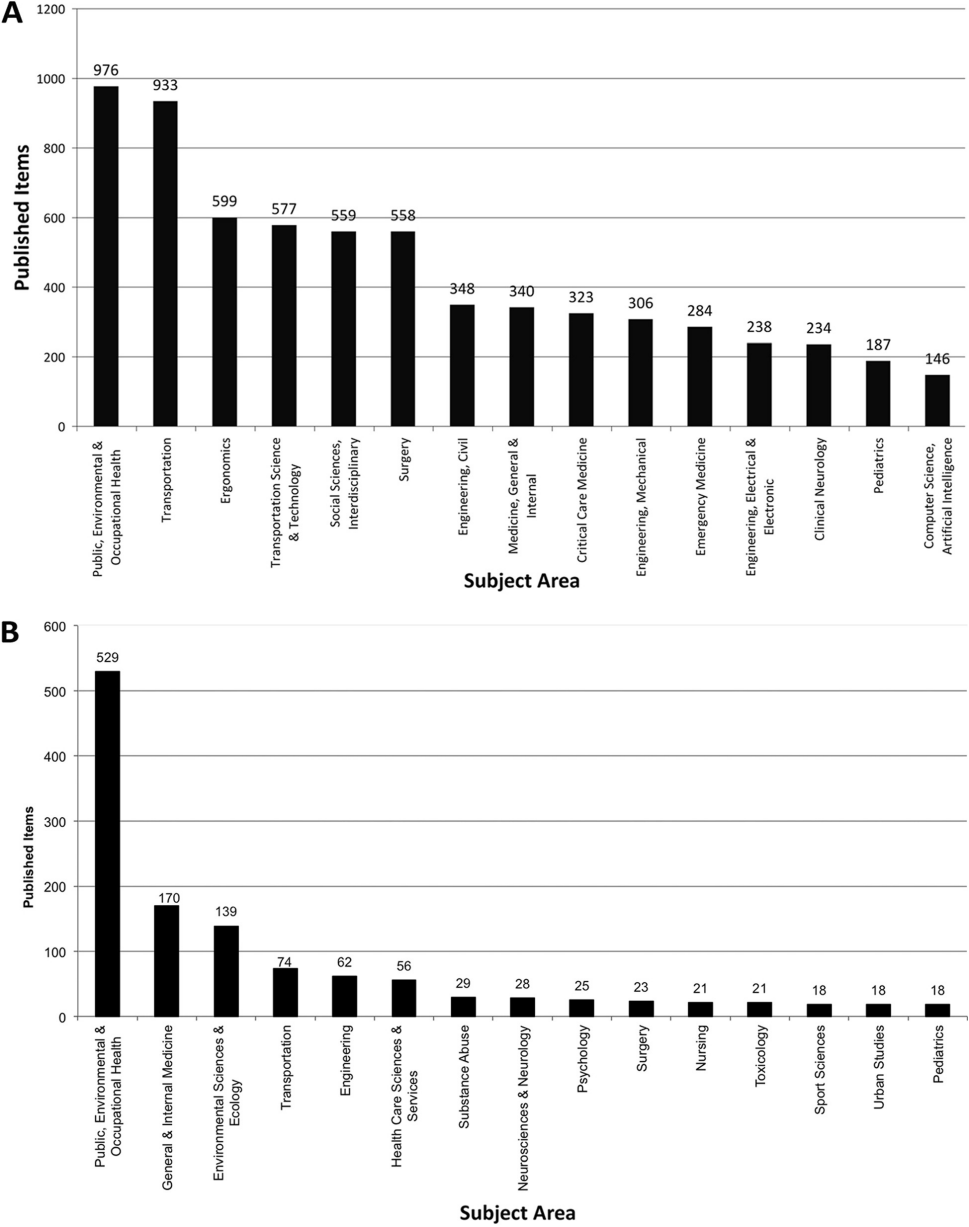
**Subject areas A:** General search: Top 15 subject areas by number of published items assigned to them **B**: Public health search: Top 15 subject areas by number of published items assigned to them.

**Figure 9:**
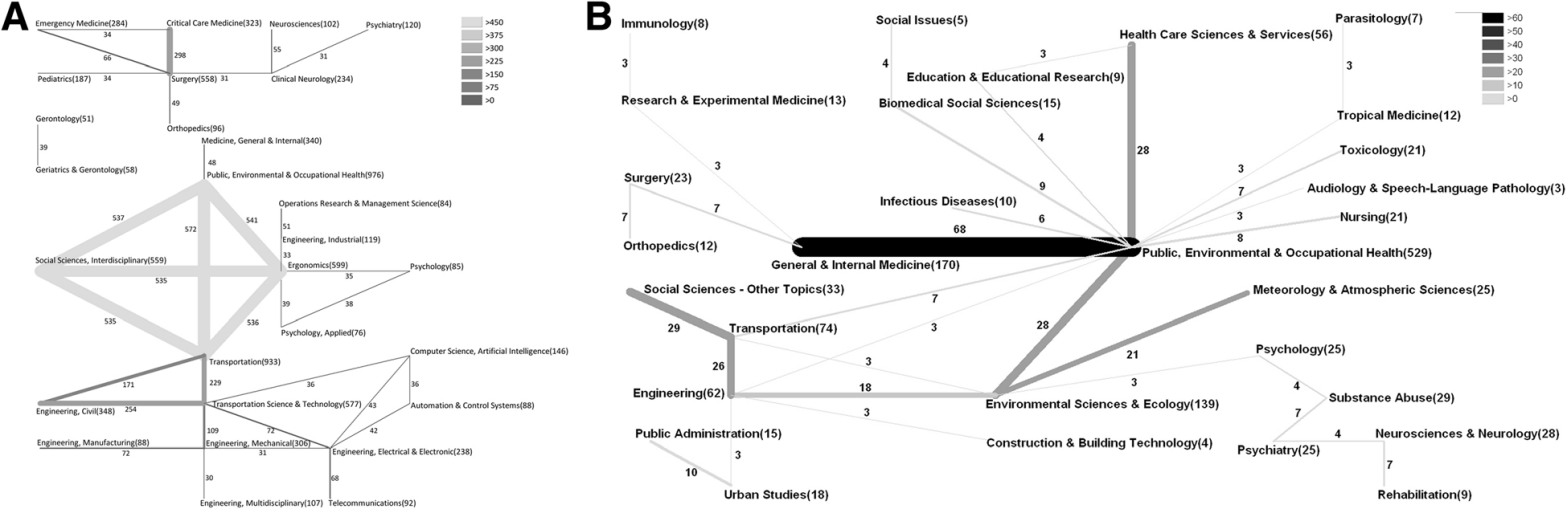
**Assignment of subject areas to articles A General search: A threshold of 30 published items was set for readability purposes.** Greyscale and thickness show amount of linking **B** Public health search. Greyscale and thickness show amount of linking.

The publications in the WoS database are also categorized concerning the subject area to which they belong. The subject area with the highest number of assignments in the general search was “Public, Environmental & Occupational Health” (976 published items), followed by “Transportation” (933), “Ergonomics” (599), “Transportation Science & Technology” (577) and “Social Sciences, Interdisciplinary” (559) (Figure 8A). When focussing on the combination of subject areas assigned to a publication, the highest number of combined subject areas was “Public, Environmental & Occupational Health” together with Transportation (Figure 9A).

In the public health search, the leading subject area was also “Public, Environmental & Occupational Health”, followed by “General & Internal Medicine” (Figure 8B). A combination of these subject areas was also the highest number of dual assignments in the public health search (Figure 9B).

### Subject area analysis - research and country focus analysis

**Figure 10:**
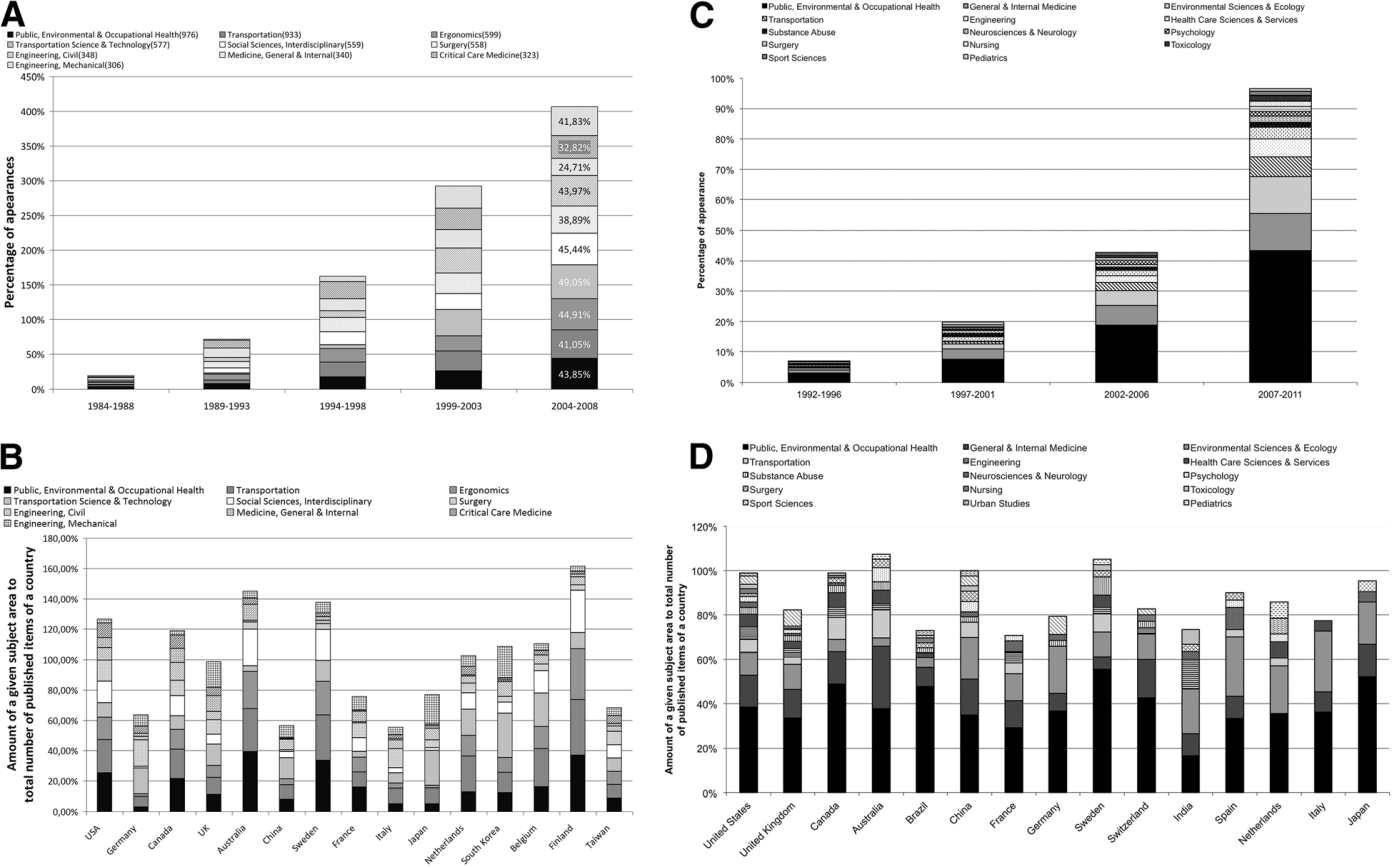
**Research areas A** General search: Research areas evolution in 5 year steps **B** General search: Distribution of the Top 10 subject areas by number of published items in the Top 15 countries by number of published items **C** Public health search: Research areas evolution in 5 year steps **D** Public health search: Distribution of the Top 10 subject areas by number of published items in the Top 15 countries by number of published items.

Analysis of the evolution of the areas of research in 5 years steps showed that in the last 5 year period monitored, the highest increase in the general search was registered in the field of “Transportation Science & Technology” with 49.05% of all 577 items being published in the period 2004–2008 (Figure 10A). Looking at the Top 15 countries by number of published items, in the USA the highest number of published items (25.28%) was assigned to the subject area “Public, Environmental & Occupational Health”, closely followed by “Transportation” with 21.85% and “Ergonomics” with 14.89% (Figure 10B). The scale of the figure surpasses the 100% because of multiple assignments of subject areas to a single article. In Germany (the second country by the number of published items), most of the research concentrated on “Surgery” (17.45%), followed by “Engineering, Electrical & Electronic” (10.40%, not included in the graphic because the subject area was ranked 12th), and “Transportation Science & Technology” (17.11%). Canada had most of its research concentrated in the field of “Public, Environmental & Occupational Health” (21.51%), followed by “Transportation” with 21.85%. In the UK most research was conducted in the field of “Engineering, Mechanical” (16.89%), followed by “Transportation Science & Technology” (14.16%) and “Transportation” (11.42%). In Australia the highest percentage was reached by the area “Public, Environmental & Occupational Health” (39.27%). “Transportation” (28.27%) ranked second followed by “Ergonomics” (24.61%).

In the public health search, a different setting was present with a more general domination of “Public, Environmental & Occupational Health” (Figure 10C, D).

## Discussion

BMC Public Health has published a number of studies related to scientometric analyses ([16,22,23]). The present study assessed the field of research related to traffic medicine using a combination of bibliometric tools and novel visualizing techniques. In the general search we found 5193 published items related to traffic medicine and starting with 1990 a dramatic increase in the number of published items was present. This is partially caused by the fact that starting with 1990 the WoS records also contained the abstract of the articles which impacts on the search routine. Another explanation could be the rapid development of cheap and fast communication means (email, Internet).

Some methodological issues need to be discussed before interpreting the data: One major issue is represented by difficulties in the terminology: In this respect, it needs to be stated that traffic medicine and related road traffic research is by definition multi-sectorial which spans both transport and health care sectors.

Given that road traffic-related research is multi-sectorial, it seems justified that many of the search terms used to identify the relevant research output focused on areas other than public health (e.g. traffic security, transport accident, transport crash etc.). At the same time, there appears to be a lack of search terms that focus specifically on the public health aspect of road traffic. Therefore, a second analysis was aimed to specifically address these aspects. Of course, also this second analysis is not all-encompassing but narrowly focussed. For instance, special issues including pedestrians, bi- and tri-cyclists are not captured completely in this approach. One therefore needs to emphasize that the present study cannot claim to exclusively focus on traffic medicine alone or transport research alone, but represents to some extent a juxtaposition.

With regard to the total output of research it can be stated that the field of traffic medicine research continues to increase annually but is relatively low in comparison to other research areas. This conclusion might be drawn when focussing on a recent study that addressed the research fields of cardiology and respiratory medicine in Europe [18]. Here, it was shown that the output levels are generally higher in these fields of medicine.

The present findings may provide useful information for those who are tasked with improving the research performance in this area. In this respect, it was recently hypothesized that without the use of scientometric techniques, there will be a growing discontent among scientists for funding allocation policies [17,18]. Therefore, the use of specific tools and benchmarking systems as shown here could be of help to implement transparency within funding allocation processes. This was the basis for the establishment of NewQIS, a scientific, non-financial platform that assesses research trends in socioeconomic important areas of science and research within delineated research projects [17,18].

Since it is not possible to precisely analyze the global funding in this area, we cannot provide any information about funding. Therefore, the assumption the published articles represent associated research funding in this area is not justified. By contrast, it is generally accepted that the biomedical fields with the highest level of funding are represented by cardiovascular medicine, oncology and the nervous system. In countries such as the US, the UK or Germany, these fields also generate the highest output. The reason is probably simple: Investing more money in one field means generating new research positions and new projects which leads to an increased number of scientific publications in this field. However, as stated above, precise models and ratios of this relation do not exist.

When relating the present study to other fields of science, previously published NewQIS studies may be used for comparison. I.e. a recent study focussed on a scientometric analysis and combined density-equalizing mapping of environmental tobacco smoke (ETS) research [24]. 6,580 ETS-related studies published between 1900 and 2008 were identified in the ISI database and a continuous increase of both quantitative and qualitative parameters was found. The combination with density-equalizing calculations demonstrated a leading position of the United States (2,959 items published) in terms of quantitative research activities [24]. Charting techniques demonstrated that there are numerous bi- and multilateral networks between different countries and institutions in this field [24]. Again, a leading position of American institutions was found. Interestingly, there are large similarities concerning the findings on country research productivity present between traffic medicine and ETS-related research [24].

However, also differing results were recently reported [8]. I.e. one study that examined specific areas of major research activity using different organ systems reported major differences between countries. In a total of 5,527,558 published items. A dichotomy was present between Western countries such as the US, UK or Germany and Asian countries such as Japan, China or South Korea concerning research focuses [8].

With regard to the presently performed search routine in the general search, we tried to assess a large number of published items by enlarging the search terms using the Boolean operator, 'OR', with different terms related to traffic medicine including "traffic* securit*", "car crash*", "car accident*", "transport* accident*", "transport* crash*", "traffic* safet*", "car safet*", "vehicle crash*", "vehicle safet*". This is in contrast to other studies that used only one search term. However, since the used search terms can be summarized into the field of traffic medicine, the present approach appears to be useful in this area of research. Undoubtedly, our search strategy encompasses a number of articles that does not represent the overall number of studies dedicated to the field of traffic medicine. This is due to the inability of every bibliometric database study to identify all published items related to a specific field within a set of over 11,000,000 publications. We therefore performed a second search that specifically addressed the issue of public health and transport/traffic medicine. In this analysis more than 1,200 specific publications were identified. In general, the trends of the first, larger analysis were paralleled concerning key benchmarks such as general output figures, leading countries or networks.

In summary, it needs to be stated that traffic medicine is a multi-sectorial research area with important implications for public health. Research related to this field of science and medicine continues to increase annually but is relatively low in comparison to other research areas. The present data may provide helpful information for those who are tasked with improving research performance in this field.

## Competing interests

The authors declare that they have no competing interests.

## Authors’ contributions

BGK, DK, SZ, CS have made substantial contributions to the conception and design of the study, acquisition of the review data and have been involved in drafting and revising the manuscript. All authors have read and approved the final manuscript.

## Pre-publication history

The pre-publication history for this paper can be accessed here:

http://www.biomedcentral.com/1471-2458/13/541/prepub
